# Maladie de Rendu-Osler: un diagnostic à ne pas méconnaitre

**DOI:** 10.11604/pamj.2015.22.40.7871

**Published:** 2015-09-17

**Authors:** Madiha Mahfoudhi, Khaled Khamassi

**Affiliations:** 1 Service de Médecine Interne A, Hôpital Charles Nicolle, Tunis, Tunisie; 2 Service ORL, Hôpital Charles Nicolle, Tunis, Tunisie

**Keywords:** Maladie de Rendu-Osler, télangiectasies cutanéo-muqueuses, héréditaires, Hereditary hemorrhagic telangiectasia, mucocutaneous telangiectasia, hereditary

## Image en medicine

La maladie de Rendu-Osler ou télangiectasies hémorragiques héréditaires est une maladie vasculaire héréditaire, rare mais ubiquitaire. La lésion élémentaire est une dilatation des vaisseaux distaux, qui se manifeste par une tendance hémorragique quand elle est cutanée ou muqueuse. Sa gravité tient à l'existence de possibles malformations artério-veineuses viscérales, en particulier pulmonaires, hépatiques et neurologiques, dont les complications peuvent être redoutables et qu'il convient donc de dépister systématiquement. Homme, âgé de 78 ans, ayant des antécédents familiaux d’épistaxis chronique non explorée, avait depuis l’âge de 45 ans des épisodes d’épistaxis récidivante, bilatérale, spontanée et de faible abondance isolée. L'examen physique a objectivé des lésions cutanéo-muqueuses à type de télangiectasies au niveau des fosses nasales, de la pointe de la langue, des lèvres, du palais, de l'oropharynx, des pulpes des doigts ainsi qu'au niveau du cavum et du larynx vues à l'endoscopie nasale souple. Le reste de l'examen oto-rhino-laryngologique, l'examen neurologique, abdominal et pulmonaire étaient normaux. L'examen biologique a révélé une anémie hypochrome microcytaire ferriprive (hémoglobine à 8.8 g/dl), un bilan d'hémostase et un bilan hépatique normaux. L'IRM cérébrale et médullaire était normale. L'angio-scanner thoracique a révélé une malformation artério-veineuse pulmonaire. L’échographie-doppler hépatique et l’échographie cardiaque étaient normales. L'endoscopie digestive haute et basse a objectivé des lésions d'angiodysplasie diffuses oeso-gastroduodénales. Au cours de l'hospitalisation, le patient a reçu un traitement martial et a présenté quelques épisodes d’épistaxis de faible abondance ne nécessitant aucune prise en charge spécifique.

**Figure 1:**
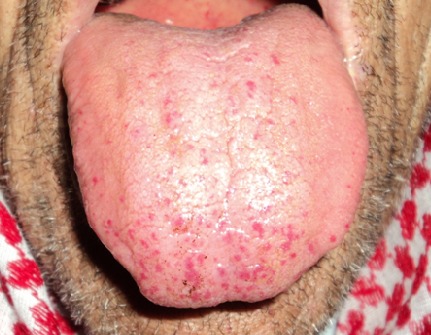
Télangiectasies cutanéo-muqueuses au niveau de la pointe de la langue, de la lèvre supérieure et du palais

